# A Review on Bacterial Contribution to Lignocellulose Breakdown into Useful Bio-Products

**DOI:** 10.3390/ijerph18116001

**Published:** 2021-06-03

**Authors:** Ogechukwu Bose Chukwuma, Mohd Rafatullah, Husnul Azan Tajarudin, Norli Ismail

**Affiliations:** Division of Environmental Technology, School of Industrial Technology, Universiti Sains Malaysia, Gelugor 11800, Penang, Malaysia; ogechukwuma@student.usm.my (O.B.C.); azan@usm.my (H.A.T.); norlii@usm.my (N.I.)

**Keywords:** anaerobic degradation, aerobic degradation, biodegradation, bio-refinery, lignocellulases, lignocellulolytic, extremophiles

## Abstract

Discovering novel bacterial strains might be the link to unlocking the value in lignocellulosic bio-refinery as we strive to find alternative and cleaner sources of energy. Bacteria display promise in lignocellulolytic breakdown because of their innate ability to adapt and grow under both optimum and extreme conditions. This versatility of bacterial strains is being harnessed, with qualities like adapting to various temperature, aero tolerance, and nutrient availability driving the use of bacteria in bio-refinery studies. Their flexible nature holds exciting promise in biotechnology, but despite recent pointers to a greener edge in the pretreatment of lignocellulose biomass and lignocellulose-driven bioconversion to value-added products, the cost of adoption and subsequent scaling up industrially still pose challenges to their adoption. However, recent studies have seen the use of co-culture, co-digestion, and bioengineering to overcome identified setbacks to using bacterial strains to breakdown lignocellulose into its major polymers and then to useful products ranging from ethanol, enzymes, biodiesel, bioflocculants, and many others. In this review, research on bacteria involved in lignocellulose breakdown is reviewed and summarized to provide background for further research. Future perspectives are explored as bacteria have a role to play in the adoption of greener energy alternatives using lignocellulosic biomass.

## 1. Introduction

Lignocellulosic biomass refers to affordable [1], superabundant, and green materials, that are vital in the goal of clean and alternative energy production and ensuring our reliance on fossils is minimized/eliminated [2]. They can be used in the sustainable production of useful chemicals, fuels [3], and renewable energy [4]. Studies have shown that lignocellulosic biomass can be broken down by contributions from several microorganisms [5] with manifold fungal and bacterial genera giving rise to cellulolytic and hemicellulolytic enzymes [6] under aerobic and anaerobic surroundings to achieve this [7]. The use of enzymes from microbial sources is being preferred to others because they are not difficult to nurture and manipulate for desired yields when compared to other sources [8]. Bacterial and fungal organisms are considered the most available biological organisms present in nature, with the ability to breakdown both manmade and natural polymers [9].

In lignin breakdown, fungi, especially white rot, have been extensively studied [10]. Despite the large amount of research that has established fungi as primary decomposers, their genetic manipulation capabilities for genetic engineering still remain very low as opposed to other organisms of interest. Fungal enzymes also do not show significant specificity and are costly to manufacture industrially. They also do not do well in extreme conditions as they cannot tolerate or adapt to altered environmental conditions [11]. Bacteria and the enzymes they produce have shown that they can adapt better to pH and temperature changes, as opposed to fungi [12]. Recent analysis of a synthetic microbial community showed that bacteria in the overall structure are more significant to lignocellulolytic enzyme activity than fungi [13]. Bacterial Glucuronoyl esterases (GEs) which arise from carbohydrate esterase family 15 were biochemically characterized and structurally determined on model substrates deepen the knowledge on their biological roles and functions and the results revealed few enzymes with higher catalytic efficiencies than previously characterized fungal GEs [14]. A halotolerant lignocellulose degrading microbial consortia was produced from salt marsh soil microbiome using wheat straw as carbon and energy source. The consortium showed bacteria possess a unique role in the breakdown of recalcitrant lignocellulose under saline conditions, as opposed to fungi. The final consortia showed greater lignocellulolytic haloenzymes than the initial one, with the results affirming bacteria’s more central role in lignocellulose degradation in saline environment compared to fungi [15].

In recent times, research focus has been turning to bacteria for lignocellulose conversion to useful products as a result of their ability and versatility [16]. To achieve success in degradation of lignocellulose biomass in the production of biofuels, it is necessary to have novel and efficient enzyme mixtures, consortia of microorganisms, and appropriate use of bioengineering to improve promising strains and create microbial communities that can use synergistic relationship with each other to breakdown the biomass. It is interesting that, in nature, bacterial abundance increases when simple carbon sources are diminished leaving their complex counterparts and higher lignin levels. These reasons, as well as a functional diversity, a wide range of terminal electron acceptors and the lignin degrading abilities are responsible for the increased bacterial interest in future biotechnological strategies [17]. A lot of research on lignin degrading bacteria has come from guts of insects and Alphaproteobacteria, Gammaproteobacteria, and Actinomycetia are some of the bacteria recognized for breaking down lignin. Discovering novel bacterial lignin degrading enzymes is vital to industrial production of next-generation biofuels. This is largely due to bacteria’s potential for use as engineered organisms involved in biofuel generation, their flexible oxygen demands and ability and range in extreme environmental conditions [18]. Time’s effect on the synergism that exists among lignocellulolytic enzymes involved in hydrolysis showed aerobic bacteria are one of the groups with the prevalent mechanism for the breakdown of lignocellulosic biomass via the free enzyme system. Anaerobic bacteria instead use an alternative lignocellulolytic system that makes use of complex protein structures like cellulosomes and xylanosomes which are supporting enzymes when biomass is hydrolyzed [19]. Bacteria also possess a handful of characteristics that make them more advantageous in the production of hydrolytic enzymes, which are vital to the degradation of lignocellulosic biomass [20]. Research findings from several researchers shows bacteria-driven breakdown of lignocellulosic biomass with some identified bacteria including *Acetovibrio*, *Bacillus*, *Bacteroides*, *Cellulomanas*, *Clostridium*, *Erwinia*, *Microbispora*, *Ruminococcus*, *Streptomyces*, and *Thermomonospora* [21].

**Figure 1:**
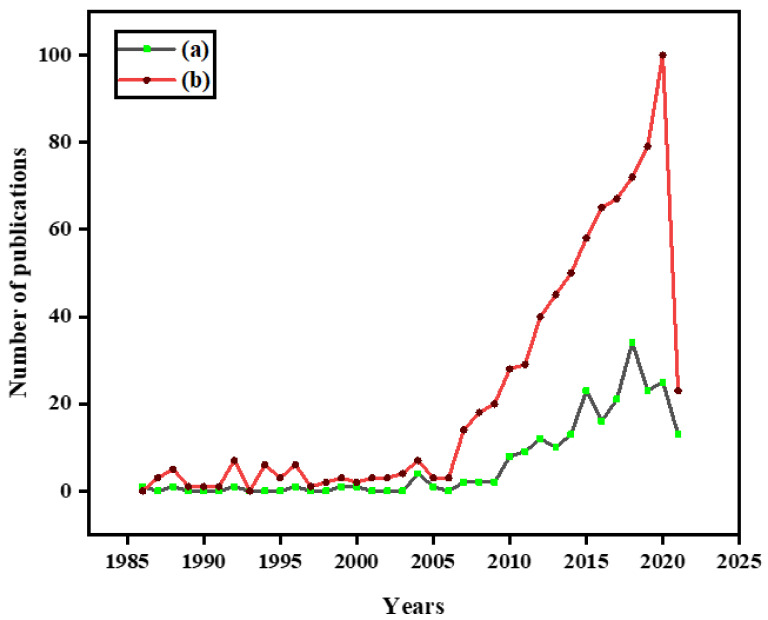
Research publication status, an analysis of yearly publication: (**a**) lignocellulolytic bacteria; (**b**) lignocellulose degradation by bacteria.

This paper covers a review of bacterial involvement in lignocellulose breakdown into useful products. A Scopus search on 6 March 2021 using the keywords “lignocellulolytic bacteria” yielded 233 papers and covers papers from 1986 till the search date. Our results showed that papers between 2015–2021 represent triple the number of papers from 2011–2014 which was 46. Another search using the key words “lignocellulose degradation by bacteria” yielded 781 papers from 1984 till the search date. As with the first search, publications increased from the early 2000s with over 600 of the papers being published since 2010. The relevance of this study can be seen in the significant rise in publications about lignocellulolytic bacteria in recent years, as depicted in Figure 1. From the literature, many articles on lignocellulose breakdown and mechanisms exist, but a focus on bacteria has only begun in recent years. Bacteria has not received as much reporting in bio-refinery applications as fungi. Due to its enormous potential, a review on available research is necessary to stimulate further research and discussion. This review captures the mechanism of delignification and cellulose in bacteria; distribution of lignocellulose degrading bacteria, bacterial enzymes involved in lignocellulose breakdown, and bio-refinery products. Molecular advancements have provided more insight to microbial communities than was previously possible. Thus, studies of bacteria for lignocellulolytic capabilities were also reviewed. Lignocellulose driven bio-refineries will thrive from bacterial adoption in their processes, and this is the basis for this review which covers the recent trends driven by molecular technologies, challenges, and perspectives for future research.

## 2. Bacteria Mechanism for Delignification and Cellulose Degradation

Bacteria are among the first and simplest life forms on earth and are ubiquitous and vital in nutrient cycling and, in turn, maintaining earth’s balance [22]. In the process of breaking down lignocellulose, bacterial cellulases, and hemicellulases are known to have several advantages and be more effective when it comes to ease to culture, possibility of speeding up production, and boosting expression [23]. Bacterial strains generally have a short generation time which means they can be grown with ease for further use in biofuel production [10]. They can withstand environmental stress better as they are biochemically versatile, with the ability to adapt to changes in temperature, salinity, pH, and oxygen availability [20]. Bacterial growth at the latter stage of lignocellulose breakdown can increase, which is especially useful as this stage is known to have materials usually difficult to breakdown. Bacterial enzymes are known to be effective between several pH ranges and its high growth rates leads to high rates of enzymes in the same vein. Bacterial lignocellulases form multienzymatic complexes that are more suited to the complex degradation of biomass [16].

Bacteria have diverse mechanisms for degrading lignocellulosic biomass but the most common is via the free enzyme system. This free enzyme system is employed largely by aerobic bacteria while anaerobic bacteria use the complex protein structures cellulosomes and xylanosomes. These act as supporting enzymes in the hydrolysis of biomass. We also have a multicatalytic enzyme system that can attain the combination of several abilities in a single gene [19]. Microorganisms involved in the breakdown of cellulose are responsible for the biggest terrestrial flow of carbon in the biosphere [24]. The most isolated cellulolytic bacteria come from the order Actinomycetales (phylum Actinobacteria), with the corresponding anaerobic order being Clostridiales (phylum Firmicutes). Cellulose degrading capability in bacteria is more rampant in Actinomycetales, which are aerobic, and Clostridiales, which are anaerobic. Anaerobic Clostridia cannot infiltrate cellulosic material and will rather proliferate on the surface of the material, releasing complex cellulases that breakdown cellulose. Lignin, which is the second most available bio-macromolecule behind cellulose [25], is a major hindrance to lignocellulose breakdown. Bacterial delignification has long been identified in *Streptomyces* belonging to the Actinomycetales order. Ligninolytic bacteria have been isolated from various environments, thus are said to be distributed all through nature. Some distinct kind of bacteria can breakdown the carbohydrate and lignin in lignocellulose. *Thermobifida fusca*, an aerobic thermophile is an example. It contains both ligninolytic and cellulolytic enzymes which it uses for lignin modification and cellulose hydrolysis respectively. Other examples are *Clostridium thermocellum* and *Caldicellulosiruptor bescii*, noteworthy bacteria that breakdown lignin while relying on sugars liberated as energy source [26]. Actinomycetia, as soil microorganisms, are heavily involved in environmental recycling achieved by the action of hydrolytic enzymes. They assist in achieving soil biotic equilibrium through their involvement in nutrients cycling [27]. The bacteria capable of degrading lignified cell walls do so in three ways: tunnelling, erosion, and cavitation [28]. The breakdown of lignin by bacteria such as Actinobacteria and Proteobacteria involves lignin depolymerisation, aromatic compound catabolism and specific product biosynthesis [29]. Lignin depolymerisation differs from that of cellulose and hemicellulose as it involves a redox reaction which consists of an electron transfer and redox potential [29]. *Streptomyces*, *Sphingomonas*, *Pseudomonas*, *Rhodococcus*, and *Nocardia* have been studied for their ability to break down lignin. Research shows bacteria use several pathways to breakdown lignin to synthesize bio-products [30].

In 1985, Ljungdahl and Eriksson [31] wrote extensively on cellulolytic bacteria and observed a surge in research interest of anaerobic cellulolytic bacteria mainly because of their potential industrial use and that they are a valuable source of cell protein. The cellulolytic bacteria’s anaerobic system in the rumen is well studied, mainly due to cellulose being a major part of an animal’s diet. Clostridium belongs to a cellulolytic bacterial group that breaks down lignocellulose in anaerobic conditions. They produce multienzyme complexes and are good producers of the cellulase enzyme. Other cellulolytic groups include *Clostridium*, *Cellulomonas*, *Bacillus*, *Thermomonospora*, *Ruminococcus*, *Bacteriodes*, *Erwinia*, *Acetovibrio*, and *Microbispora* [32].

**Figure 2:**
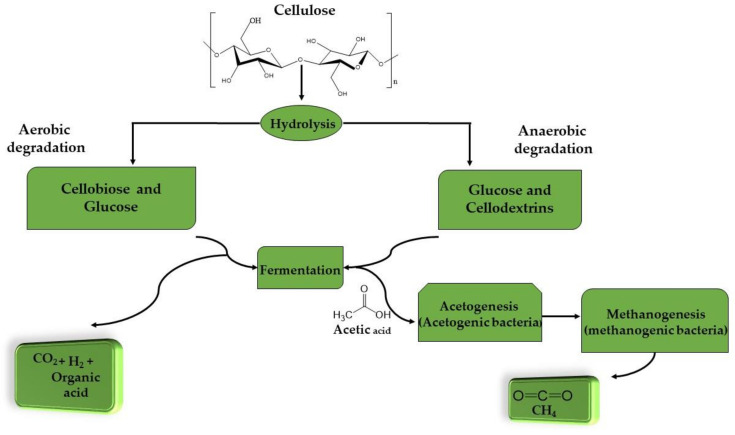
Aerobic and anaerobic degradation of cellulose showing the primary and secondary by-products.

Aerobic biodegradation means the breakdown of organic matter by microorganisms in the presence of air, usually in a moist and warm environment [33]. Figure 2 shows both aerobic and anaerobic degradation of cellulose. Aerobic bacteria break down cellulose by releasing free cellulolytic enzymes which act on the biomass [34]. Primarily, the bacteria hydrolyze the cellulose and convert it into cellobiose, then fermentation, which refers to the hydrolysis of the cellobiose, occurs and this produces carbon dioxide, hydrogen, and organic acids [35]. After this stage, the dominant bacteria utilize these secondary products to produce various useful products [36].

Anaerobic biodegradation is the breakdown of organic matter by microorganisms without the presence of oxygen via several metabolic connections [33]. When bacterial anaerobic fermentation of biomass occurs, sugars are converted to alcohol or acids, then biogas results from anaerobic digestion of the acids or alcohols [35]. Microorganisms that use cellulose come from several groups, which include methanogens and acetogens. CO_2_ is the main product of microbial action on cellulose but in anaerobic conditions, methane CH_4_ is also produced. Cellulolytic microorganisms (primary microorganisms) begin the degradation process of cellulose and the primary products are sugars, which are used for growth and maintenance. Then naturally we have the secondary microorganisms, which are unable to directly hydrolyze cellulose but use the products of the actions of the primary microorganisms for survival. They form symbiotic relationships that see the secondary microorganisms removing the free sugars produced by the primary ones, which would otherwise hinder further cellulose degradation. This “symbiosis” is what drives cellulose degradation. In aerobic environments, this association between the primary and secondary microorganisms suffices for the absolute oxidation of cellulose to CO_2_ [31]. While the general belief is that cellulose is broken down in an aerobic environment, studies have shown that it can also happen in an anaerobic environment. The products depend on the peculiar anaerobic habitat, in rumen, cellulose is converted to acids like acetate, butyrate and propionate, which the animal needs. In other anaerobic habitats such as digestors, CH_4_ and CO_2_ are the products. Cellulose breakdown without oxygen involves two interrelated processes:

- Cellulose being oxidized to form CO_2_ and protons being concurrently reduced to H_2_;
- H_2_ being oxidized as CO_2_ is reduced to acetate and then methane, sulfate to hydrogen sulphide or nitrate reduced to ammonia.

H_2_ is vital to the anaerobic breakdown of cellulose as the first step requires the bacteria involved to produce H_2_, while the next step needs bacteria that consume H_2_ [31].

## 3. Distribution of Lignocellulolytic Bacteria

Extremophiles are microorganisms that can endure and adapt to aggravated environmental conditions such as high or low salinity, temperature, pressure, or pH. They are crucial in the bio-refinery process as the biocatalysts they produce can be applied in industrial and biotechnological processes [37]. Thermophilic bacteria (Thermophiles) refers to microorganisms that crave heat and grow optimally in temperatures above 45 °C. They can be found in deep sea hydrothermal vents, terrestrial hot springs, volcanic sites compost, and deep organic landfills [37]. The rising interest in thermophilic bacteria is largely because they possess distinct cellulolytic and hemicellulolytic systems and are a possible source of potent and thermostable enzymes for use in biomass hydrolysis. They are also involved in the fermentation of a wide array of carbohydrates into ethanol, with some showing high yields. The development of genetic tools and studies has allowed them to be bio-engineered leading to high ethanol yields as they are now involved in second generation production of ethanol geared to reduce costs [38]. Thermophiles are either extreme thermophiles (65–79 °C), moderate thermophiles (45–64 °C), and hyperthermophiles (temperatures higher than 80 °C) [39]. Thermophilic microorganisms sourced from marine hot springs and hydrothermal vents are producers of polymers and enzymes which can be used industrially [40]. Bacteria sourced from compost and soil were studied for their lignocellulolytic and thermophilic properties. Thermophilic isolates (*Paenibacillus validus*, *Paenibacillus koreensis*, *Paenibacillus thailandensis*, *Paenibacillus cellulositrophicus*, *Paenibacillus lautus*, *Bacillus nealsonii*) were found to have high potency of lignocellulose enzymes ligninase and cellulase [41].

Mesophiles grow at temperatures between 20–45 °C but have an optimum temperature range of 30–39 °C. They are found in soil and water environments and are known to be involved in biodegradation of organic matter [42]. Both mesophilic and thermophilic organisms partake in degradation of lignin found in lignocellulose substrates. Mesophiles degrade easily degradable biomass and this causes the temperature in the environment to increase. Some biomass with high lignocellulose fractions resists bacterial degradation under mesophilic conditions. However, when temperatures are elevated, thermophilic organisms are able to break these high lignocellulose fraction thus releasing them for microbial degradation [43]. A mesophilic microbial consortium was studied for ability to degrade a potent crop, Napier grass and showed promise to enhance the efficiency of using Napier grass and other cellulosic wastes to produce bioenergy via anaerobic digestion [44].

Psychrophiles grow at temperatures below 20 °C and are found in the cold regions like the Antarctic. They are also in glaciers and freezing appliances. Psychrophilic refers to those permanently cold whereas psychrotrophic refers to those who are seasonally cold, usually in areas that fluctuate between psychrophilic and mesophilic. Psychrophilic microorganisms have shown promise in industrial applications and their enzymes, psychrozymes, thrive at temperatures below 15 °C. This ability to function in the cold makes them advantageous in biotechnology, particularly biofuels and energy production. Psychrozymes break down lignocellulose at these very low temperature conditions, have reduced energy requirements, and lesser chemical requirements than breaking down lignocellulose at higher temperature conditions [45]. The first report of wood degradation in the Antarctic reveals lignocellulolytic microorganism that degraded wood cells of subject materials, submerged for a long period. The study confirmed that lignocellulolytic bacteria are highly active even in such an extreme environment [46]. Bacteria can sometimes be versatile and exist in less than optimal temperatures. An anaerobic microbial consortium TC-5 was studied to determine if it could improve lignocellulose-degradation of un-pretreated wheat straw, and the results show the multiple species in the consortium contain lignocellulolytic enzyme that improved yield at both mesophilic and thermophilic temperatures [47].

Alkaliphiles are extremophiles that can survive in alkaline environments with pH range 8.5 to 11. Enzymes from alkaliphiles are advantageous in biofuel production, as they are known for high thermostability. Acidophilic bacteria survive in acidic environments with pH 1 to 5. Their high membrane impermeability and membrane potential reversal are some of the functions that make them good prospects in industrial applications [45]. Barophilic microorganisms can live in environments with irregular pressure levels. Piezophiles are those microorganisms that can grow in conditions of higher than atmospheric pressure, while hyperpiezophiles are those that can grow at pressures higher than >50 MPa [40]. Halophiles are microorganism that grow optimally in saline conditions. There are also halotolerant organisms that can grow in highly saline conditions and normal conditions as well. Lignocellulolytic enzymes like cellulase, xylanase, and laccase, used in the production of biofuel, when derived from halophilic and halotolerant sources are very stable [45].

## 4. Classification and Production of Bacterial Lignocellulases

Microbial communities generally consist of organisms that produce various enzymes that specifically breakdown certain substrates. This pack of enzymes are useful in lignocellulose driven bio-refineries [48]. Biological enzymes are an economical, green, more efficient option which uses little energy in the production process. These reasons account for the massive attention they are now receiving [49]. Cellulose degrading enzymes degrade biomolecules into their simplest forms and they include cellulases, hemicellulases, pectinases, chitinases, amylases, proteases, esterases, and mannanase. Also referred to as hydrolytic enzymes, they are of commercial importance as they make up a large part of the global enzyme market due to their rising use industrially and biotechnologically [50]. Apart from producing various hydrolytic enzymes, a number of microorganism are capable of producing several isozymes of the same enzyme [6]. The bacterial enzyme system when lignocellulose is degraded can be a non-complexed or complexed system synonymous with aerobic and anaerobic bacteria, respectively [51]. The complexed system, driven by anaerobic bacteria, is said to have greater potential in hydrolysis of biomass during the breakdown of lignocellulose biomass [52]. Having a more robust understanding of how enzymes interact will assist in developing more efficient consortium that can be used in lignocellulose breakdown. Lignocellulolytic enzymes, such as amylases, (hemi)cellulases, chitinases, and pectinases, can be used for industrial purposes and also in bio-refineries. Bacteria that can produce extracellular enzymes in varying temperature and pH conditions are very useful for industrial and biotechnological applications. Some bacteria genera that are known extracellular enzymes producers are *Pseudomonas*, *Enterobacter*, *Bacillus*, *Klebsiella*, *Paenibacillus*, *Rhodococcus*, *Cellulomonas*, *Streptomyces*, and *Citrobacter* [12].

Bacillus species are a group of thermotolerant species that have shown lignocellulolytic properties due to the production of lignocellulases. *B. subtilis* has shown potential in rice straw composting in a study where it had the highest expression of Lignin peroxide (LiP) and cellulases [53]. *Bacillus* sp. R2 was used to produce lignocellulolytic enzymes from spent coffee after different forms of pre-treatment. The results showed the best inducers for cellulase and pectinase was spent coffee grounds without any treatment, as pretreatment removed certain materials used by the bacterial community for enzyme production [54]. *Bacillus* sp. *BS-5* showed potentials in producing a cost-effective and efficient lignocellulolytic enzyme cocktail (xylanase and endo glucanase) that can be used in saccharification of lignocellulose biomass as the results with corn cob as a substrate was promising [55]. *Bacillus* sp. 275 showed lignocellulolytic enzyme expression of cellulase, xylanase, laccase, and peroxidase in its cell culture supernatant [56]. Actinobacteria produce lignocellulolytic, oxidative, and different industrially useful enzymes that can be used for biotechnological applications. They produce extremophilic enzymes, which are biocatalysts produced by microorganisms in stressful conditions such as unideal pressure, temperature, pH, and salinity. These extremophilic enzymes can withstand and function in extreme conditions. Actinobacteria also produce laccase and peroxidase that are oxidative enzymes with catalyzing properties that are used in many green industry processes [57]. *Caldicellulosiruptor* species are known as glycoside hydrolase (GHs) producers that have the ability to degrade cellulose under extreme temperature of 70 °C. Cocktails of GHs from three *Caldicellulosiruptor* species were mixtures of enzymes that showed greater promise than single enzymes in breaking down microcrystalline cellulose [58].

## 5. Common Substrates for Lignocellulose Based Bio-Refineries

Lignocellulosic biomass is sometimes classified as plant biomass that falls under: agricultural wastes, hardwood, softwood, and grasses [59]. Lignocellulosic materials such as bagasse, straws, stalks, and cobs are seen as alternative raw materials for bio-refineries [60]. Bio-refining’s success in terms of cost is linked to availability of the right feedstock as a feedstock that is sustainable at low cost is needed to reduce its cost. An alternative classification means grouping lignocellulosic biomass as agricultural waste, forestry residue, urban waste, industrial waste and energy crops. Energy crops are agricultural crops like corn, cassava, sweet sorghum and switch grass. While agricultural residues are wheat and paddy straws, corn stover and cobs, shells, stalks, hulls, discarded fibre, bagasse, and husks. Forest residues are dead branches, prunings, slashes, and wood chips. Urban and industrial waste are fruit and vegetable processing waste, linters, household waste [61]. Straws from rice, barley, and wheat are lignocellulosic substrates being used as second generation biofuel feedstock. They are readily available and cheap. They regenerate quickly and have high sugar content. Cereal straws after undergoing pre-treatment can be degraded by specific microorganisms to produce bioethanol, biobutanol, biohydrogen and biogas/biomethane [62].

It is well known that bacterial can be feedstock-specific, which may affect yield and overall system efficiency. Lignocellulosic biomass contains three major polymers and cereal residues like straws and bagasse have higher lignocellulosic content than agricultural wastes and grasses [59]. Deng [63] in 2016 compared lignocellulolytic ability of bacteria isolated from lignocellulosic salt marsh detritus and glucose media. The results show that how complex a substrate is can affect bacterial growth and interactions. Whereas complex substrates like lignocellulose induce positive interactions and synergistic growth, simple and labile substrates like glucose encourage mostly negative interactions and competition. Lignocellulose breakdown in the environment is spurred by synergistic relationships that exist between indigenous bacteria found in the environment. A review of fungal cellulases as well as bacterial hemicellulases suggested that combined cross-linked enzyme aggregates (combi-CLEAs) can be applied in the future affordable and versatile tools to improve the synergistic interactions between fungi and bacteria in lignocellulose based processes [64]. Levin et al. [65] covered the advantages of deploying cocultures of lignocellulolytic microbes over mono cultures in industrial processes. The synergistic relationship in cocultures can be exploited and used to ferment lignocellulosic substrates. This is possible by using omics technology to gain operational understanding of communities and functions and replicate them to develop designer microbial consortia for consolidated bioprocessing (CBP). CBP is seen as a cheap route to deriving bioethanol from lignocellulosic feedstock [66]. The incidence of antagonistic interactions among lignocellulolytic bacteria is brought to a minimum when carbon source is derived from complex substrates instead of simple sugars like glucose. There are less antagonistic interactions on complex media and more antagonism on simple media [67]. Production of methane using single and co-digestion with palm oil mill wastes as a substrate, *Bacillus subtillis*, and a mixed culture of methanogenic anaerobes at varying loading percentages. Results showed that *B. subtilis* had a more positive impact on the co-digestion than the mixed culture of methanogens, largely due to its cellulolytic ability displayed in the degradation of the empty fruit bunch [68]. Degradation of rice straw was done effectively by a novel psychrotrophic lignocellulose degrading microbial consortium LTF-27 made up of *Parabacteroides*, *Alcaligenes*, *Lysinibacillus*, *Sphingobacterium*, and *Clostridium* [69]. The mechanism used to convert citrus peel wastes (CPW) into bioflocculants using *Alcaligenes faecalis subsp. phenolicus* ZY-16 was analyzed. CPW can be a cost-effective feedstock used to produce bioflocculants, which have potential to be applied in microcystin removal [70]. Table 1 shows common lignocellulosic substrates, as seen in previous studies.

## 6. Bio-Refinery Products

Bioenergy comprises various forms of energy sources, whether they are liquid, gaseous, or solid. These arise from a bio-refinery which is a platform that employs the use of biomass to produces bioenergy [45]. The saccharification of lignocellulose biomass leads to a release of sugars which can be used for downstream production of useful products [48]. Biogas, bioethanol and biodiesel are the major products from lignocellulose biomass after various reactions have occurred. However, heat and electricity are also by-products of bio-refineries [116].

At the moment, bioethanol is the most well-known biofuel, already widely accepted in the US, Brazil, the European Union, and China [117]. Second-generation ethanol refers to ethanol derived from crop residues or non-food crops whose major constituent is lignocellulose. Bioethanol is produced either via biochemical or thermochemical conversion [118]. Various microorganisms convert lignocellulose into bioethanol and they contribute to the different steps of the conversion process (pretreatment, hydrolysis, detoxification, and fermentation). The group, species, and strain of the microorganism affects the bioethanol yield. A good ethanologenic microorganism should be able to grow in simple and cheap media, produce yields greater than 90%, be able to repel growth of other microorganisms which can cause contamination, and have high ethanol tolerance and productivity levels [119]. The conditions for bioethanol production are either separate hydrolysis and fermentation, separate hydrolysis and co-fermentation, simultaneous saccharification and fermentation, simultaneous saccharification and co-fermentation, pre-saccharification and simultaneous saccharification and fermentation, or consolidated bioprocessing. Consolidated bioprocessing is regarded as a very promising approach in the sustainable production of ethanol from lignocellulose with *Thermoanaerobacterium saccharolyticum*, *T. thermosaccharolyticum*, *Thermoanaerobacter mathranii*, *T. ethanolicus*, and *Geobacillus thermoglucosidasius*, and *Corynebacterium glutamicum* as bacteria already studied for bioethanol production [120].

Biogas refers to the product of anaerobic digestion of biomass and is mainly made up of CH_4_, CO_2_ [121], and N_2_ [122]. Anaerobic digestion involves conversion of biomass in the absence of oxygen and involves an array of microorganisms in different metabolic processes [123,124]. Hydrolysis is converting complex polymers to simpler amino acids and sugars by the action of hydrolytic enzymes which come from cellulolytic bacteria [6]. Temperature plays a huge role in the activity of hydrolysis. Thermophilic temperatures mean digestion occurs over a short period, but it can be unstable and have huge energy demands for completion. Its hydrolysis rate is twice that of the mesophilic range [125]. Acidogenesis and methanogenesis are performed by acetogenic bacteria (acid formers) and methanogens respectively. The former works on the by-products of hydrolysis to produce organic acids, carbon dioxide, and hydrogen, while the latter cleaves to organic acid producing methane and carbon dioxide [124].

Biohydrogen is a clean energy carrier that is a by-product of microorganisms acting on organic biomass [126]. Bacteria that produce biohydrogen are either photosynthetic, aerobic, or anaerobic [127]. *Bacillus*, *Enterobacter*, *Clostridium*, *Lactobacillus*, and *Bfidobacterium* have been used to produce biohydrogen successfully [128]. Biohydrogen can be derived from biomass by a thermal or biological process. The thermal process has a lower yield and is not very efficient. The biological process could either be photo biological process, photo fermentation or dark fermentation [129]. Dark fermentation is very flexible and does not require rigid sterile or oxygen demands [130]. Bacteria such as *Pseudomonas* and Bacilli have been known to anaerobically degrade waste oil to produce surfactants and lubricants. These are also useful chemicals that are applied as foaming agents, dispersants and emulsifiers. Biosurfactants are less toxic and more biodegradable as opposed to fossil surfactants and thus are being preferred for various bioremediation efforts and use in the cosmetic, food, and pharmaceutical industries [131].

## 7. Molecular Approaches of Bacterial Delignification and Cellulolytic Production

Molecular approaches have made a huge impact in improving the world’s understanding of lignocellulose breakdown into valuable products [5]. Mega sequencing platforms with the ability to work on billions of reads are causing revolutions and giving rise to new fields such as metagenomics, metatranscriptomics, and proteogenomics. This is allowing for studies to be performed faster and in larger proportions. Metagenomic approaches can now be used in characterizing microbial communities and the inherent active metabolic pathways. The introduction of large metagenomics data and whole-genome sequencing via metaproteomics has resulted in researchers being able to link phylogeny with how microorganisms function [37]. Metagenomics has provided an easy way to discover novel thermozymes from thermophilic and hyperthermophilic microorganisms. These processes are hinged on a primary step of extracting high quality DNA from studies involving lignocellulosic microorganisms, which is then used to explore microbial populations determining culturable and unculturable populations [132]. Molecular methods allow specific characterization of bacteria. Chien et al. [133] in 2015 used sequencing to study landfill leachate and revealed two new cellulolytic strains of the genera *Paenibacillus* and enzyme extracts with good catalytic potential. Sequencing of *Streptomyces griseorubens* JSD-1’s showed its lignocellulose-degrading ability with lignocellulolytic genes such as multicopper oxidase, exo-1, 4-β-glucanase, endo-1,4-β-glucanase, and β-xylosidase, which all contribute to the biodegradation of lignocellulose biomass [134]. Sphingobacteriales, Clostridiales, and Spirochaeta were characterized from lake sediments using both molecular and culture dependent methods. The results revealed that anaerobic alkaline habitats have diverse microbial communities capable of degrading lignocellulose are thus a possible resource for improving anaerobic digestion [135]. *Streptomyces* sp. despite being known degraders of plant biomass have only few strains that have been characterized genetically. A genomic study revealed the function characteristics of two novel strains with promising hemicellulolytic and cellulolytic properties [136].

These methods allow for scarcely-studied species to be observed and more knowledge of their lignocellulolytic potential gathered. A lignocellulose degrading bacteria from the underexplored genus *Meridianimaribacter* was studied for lignocellulolytic properties and showed promising enzymatic activity [137]. Metagenomic analysis of the bacterial community in Vietnamese native goat rumen revealed the structure and diversity of lignocellulolytic bacteria in that microbial community and an understanding of lignocellulolytic functions. Results imply that having a higher ratio of Bacteroidetes to that of Firmicutes leads to increased lignocellulose degradation [138]. Using the 16S rRNA gene, a novel lignocellulolytic bacterial species, *Chryseobacterium gleum,* was discovered after growth in a culture medium having lignin or cellulose as the sole carbon source. Quantitative and qualitative assays were then carried out to assess the bacteria’s lignocellulolytic activity. Five other lignocellulolytic bacteria *Acinetobacter* sp., *K. variicola*, *Bacillus* sp., *P. mirabilis* and *S. maltophilia* that degrade either cellulose or lignin were also isolated [139]. High-throughput 16S amplicon sequencing and silico prediction was employed to characterize bacterial communities and determine function potential. *Proteobacteria*, *Firmicutes*, and *Actinobacteria* were the most dominant genera, and there was endocellulase function [140].

Carbohydrate-active enzymes (CAZyme) refer to the group of enzymes whose actions in nature lead to enzymatic breakdown of cellulose and hemicellulose [141]. The functional stability of the CAZyme profiles in the large intestine and rectal sites of sheep was carried out. A comparison with that of cow rumen showed less of the families of enzymes that degrade cellulose and xylan [142]. Genomic study of *Pantoea ananatis* Sd-1 was carried out to reveal inherent genes for carbohydrate-active enzymes. The findings gave insight into its degradative system, as lignocellulolytic enzymes of the bacterium were identified and characterized to understand how it degrades lignocellulose and how it can be applied in producing sustainable energy [143]. Metagenomic analysis of microbial consortia enriched from compost showed CAZymes involved in lignocellulose decomposition. The results revealed thermophiles from Actinobacteria that could effectively breakdown lignocellulosic biomass found in compost [144]. Genes from *Actinomycetales* families were enumerated for CAZyme expression and showed the genes were encoding enzymes known for cellulose, chitin, xylan, and pectin degradation [91]. A full spectrum of genes that encode CAZymes mostly from *Bacteroidaceae, Ruminococcaceae, Rikenellaceae, Clostridiaceae, and Prevotellaceae* were found in the fecal community of yak during a metagenomic study [145]. Metasecretomics involves the study of the entire proteins in a bacterial community with the intention of identifying the exact proteins involved in biomass degradation. This was applied to the analysis of a microbial consortium grown in different carbon sources and the findings revealed several extracellular enzymes involved in lignocellulose degradation. This study also showed more activity in the more complex substrate [146]. Metagenome sequencing and metaproteomics were used to study EMSD5’s community structure and enzymes it expresses when grown on corn stover. Results indicated the community was largely comprised of the phyla Proteobacteria, Bacteroidetes, and Firmicutes [82].

These studies also provide insight on not just composition but succession systems in ecosystems. The composition and functional potential of fungi and bacteria communities was investigated using shotgun metagenomics and extracellular enzyme assays [147]. The microbial community in agricultural biogas fermenters was compared with natural systems to determine why the former had less hydrolytic potential despite being an avid transformer of plant biomass to methane under anaerobic conditions. The deep metagenome and metatranscriptomics results revealed that bacteria that express genes for lignocellulolytic enzymes were present in lower numbers [148]. Microbiota from lake sediment was used as the starting medium to construct three consortia which were studied anaerobically for lignocellulolytic enzyme activity. The succession study revealed that the bacterial consortium is an appealing biological tool for the depolymerization of recalcitrant lignocellulosic materials and was proposed to be used in producing precursors of biofuels [149]. Metatranscriptomics was used to study the metatranscriptomes of the higher termite symbiotic communities, with a focus on novel CAZymes capable of hydrolyzing lignocellulose biomass. The study proffered an advantageous method to study metatranscriptomic profiles of the higher termite gut symbiotic bacteria as they reveal interesting enzymes capable of hydrolyzing a broad range of chemical bonds [150]. Using metatranscriptomics, lignocellulose degrading enzymes from a five-species (*Stenotrophomonas*, *Paenibacillus*, *Microbacterium*, *Chryseobacterium*, and *Brevundimonas*) synthetic bacterial consortium was studied. The strains showed the best level of collaboration at the initial stage of growth. The results show expression of lignocellulolytic enzymes capable of improving the saccharification process of biomass [151]. Metagenome and metatranscriptome sequencing was used to study how nutrient availability affects the microbial community’s structure and functions expressed with the aim of improving biomethane production from rice straw that has not undergone any thermal pretreatment. Results showed an over 80% yield increase, providing proof that this is a greener and more sustainable option to obtain bioenergy from rice straw [105]. Jiménez et al. [152] in 2015 using metagenomics studied two matured consortia to understand their genetic make-up and role in the bio-degradative process of wheat. They found evidence that untreated wheat straw which had more lignin, hemicellulose, and cellulose when compared with heat-treated wheat was the preferred substrate. However, both microbial consortia had the ability to break down lignocellulose. Genome study of a compost consortia discovered new thermophilic bacterial species that are known lignocellulose degraders [153]. Metagenomics was used to identify Bacteroidetes, Firmicutes, Proteobacteria, Actinobacteria, Fibrobacter, Bacteroides, Clostridium, Prevotella, and Ruminococcus as the major bacteria in thr rumen microbiome and identify carbohydrate-active genes of Bostaurus (cow) and Bubalus bubalis (buffalo) that had eaten green or dry roughage. Results showed the microbiome in buffalo rumen was more efficient in hydrolysis of plant biomass [104]. Using stable isotope probing and metagenomics, *Caulobacteraceae* was found to degrade all three lignocellulosic polymers in a study to identify and characterize functional attributes of lignocellulolytic fungi and bacteria from coniferous forest soils in North America [154]. Whole genome sequencing of *Bacillus* sp. 275 showed genes in the genome with a potentially a wide range of uses in the degradation of lignocellulosic biomasses [56]. The complete genome sequence of *Bacillus velezensis* ZY-1-1 showed genes with xylanase and cellulase producing abilities using hemicellulosic and cellulosic substrates [155]. Another genome analysis of the *Bacillus velezensis* strain identified distinct genes of lignocellulolytic enzymes and predict potential to breakdown lignocellulose. Despite showing limited ability to degrade lignin, the strains however showed potential to degrade cellulose and hemicelluloses. This implies that lignin degradation will require pretreatment of substrate [156]. *Paenibacillus polymyxa* ND25 was analyzed and showed a diverse lignocellulolytic enzyme system capable of cellulose and hemicellulose hydrolysis, showing that the bacteria has huge potential for application in biogas and other value added products production [96]. The ability of bacterial strains, sourced from the bark beetle species, to breakdown plant cells was studied using draft genome sequencing and showed lignocellulolytic enzyme production and could be useful in biotechnological processes involving biomass hydrolysis and bioremediation. *Curtobacterium*, *Erwinia*, *Pantoea*, *Pseudomonas*, *Rahnella*, *Staphylococcus*, and *Yersinia* especially showed lignocellulolytic properties towards cellulose, xylan, and starch [157]. Genome mining was used in the novel study of the structures and functions of multiple-dockerin proteins in cellulosomes which play a major role in lignocellulose degradation by some lignocellulolytic bacteria, *Clostridium thermocellum DSM1313*. The chemical shift assigned here form the basis of future studies of multiple-dockerin proteins to gain more insight into structure and function [158]. As more information from molecular studies become available, it will become easier to analyze and compare samples from various environments. Questions about diversity, function, structure and the active bacterial community can gain insight and impact positively on bacterial applications in research and industry [5].

## 8. Challenges and Future Perspectives

Despite the clear benefits of deploying bacteria in the breakdown of lignocellulose to useful end products, its adoption is marred by several challenges which range from cost and difficulty in upscaling. Delignification is vital, and despite recent advances, the titer of the final bio-product remains at low levels for studies carried out on bioconversion of lignin [29]. Single cultures are not very effective in breaking down the components of lignocellulose [159]. In delignification, a single species cannot achieve the desired results, as studies have shown that even in nature the collective effort of several microorganism in aerobic and anaerobic conditions is what leads to lignin depolymerisation [160]. Whilst the isolation of pure cultures is still necessary for research and genetic engineering [159], the process needs simplification [161] and genetic modification still poses many challenges [162]. Bacteria need huge genetic engineering to improve biofuel yields and keep cost competitive [163]. Bacterial also are feedstock specific which may prove challenging as hydrolysis process relies on the availability of the biomass rather than the concentration of the enzyme [160]. The production of enzymes from bacteria requires more specificity than others [164]. Bacterial enzyme complexes are more difficult to construct and study [16]. Thorough research on bacteria regarding lignocellulose breakdown is limited or still in its infancy [28].

It is quite rare for microorganisms to display the simultaneous ability to degrade cellulose, lignin, and hemicellulose. This provides the need to continue to search for microorganisms that have better lignocellulose degrading abilities, as their applications in bio-refinery will be broad. It is expected that such new microbes will show greater efficiency at turning complex plant biomass into fermentable sugars. Any organism that can show multi-enzyme (lignocellulolytic) ability can be suitable, as it will have a broader substrate affinity [165]. *Bacillus circulans* and *Bacillus megaterium* have already shown cellulolytic and hemicellulolytic multi enzyme complexes [166] and more discovery of other microorganisms with such ability is required.

Using co-cultures of microorganisms is being viewed as an efficient method to break down cellulosic biomass for biotechnological use, with clear advantages over the use of single strains [167]. Studies using the mixed culture system showed higher yields and the more complex biomass yields more than simple carbon sources [159]. Adopting a combination of cellulolytic and non-cellulolytic microbes gave positive results in yields, aerotoerance, and overall hydrolysis [168]. Monoculture approach was compared to a co-culture (*Clostridium thermocellum*, *C. stercorarium*, and *Thermoanaerobacter thermohydrosulfuricus*) using different biomass. The co-culture showed more promise in digesting biomass [115]. Using mixed cultures to degrade lignocellulose comes from an understanding that lignin in nature is not removed by a single species. It is instead broken down by a collective-cooperative effort of several microorganism under varying environmental conditions, Using a microbial consortium removes setbacks such as regulation of feedback and repressed metabolites, which are common in treatments involving single species [160]. Positive microbial interactions that exist in microbial communities will be good models for advances in bacterial ligninolytic, cellulolytic and hemicellulolytic abilities [169]. Lignin displays less inhibition to decomposition when in an aerobic environment because of the physical association that exists between lignin and cellulose (sheathing). This makes lignin relatively more degradable in aerobic environments as opposed to the refractory behavior in anaerobic environments [170].

Isolation of pure culture is still vital despite of the advent of omics in the characterization and determining function of microbial communities. Microorganisms that can survive in both aerobic and anaerobic conditions while concurrently degrading cellulose will prove very advantageous. *Caulobacter* sp. FMC1, a facultative mesophilic cellulolytic bacterium, showed positive results in degrading cellulose both aerobically and anaerobically [171]. Co-digestion of substrates has been found to increase yields, and several studies show success in the co-digestion of lignocellulosic wastes and livestock waste [172].

Thermophilic bacteria are also gaining interest as they possess distinct cellulolytic and hemicellulolytic systems. This makes them possible sources of potent and thermostable enzymes that can be used in the hydrolysis of biomass. They are involved in the fermentation of a wide array of carbohydrates into ethanol, with some showing high yields. The development of genetic tools and studies has allowed them to be bio-engineered, leading to high ethanol yields, as they are now involved in second generation production of ethanol geared to reduce costs [38]. Bio-engineering will help in producing microorganisms capable of releasing external enzymes for lignocellulosic depolymerization, as promising genes can be combined to produce maximum effect and lower cost [29]. Developing working pretreatment techniques will require affordable lignocellulolytic enzymes, which can be sourced from improved strains. These could ensure future pretreatment is directed to ensuring both lignin and sugars from lignocellulose breakdown can be used in separate process streams to yield useful products [173].

For lignocellulosic bio-refineries to gain more acceptance and commercialization, policies need to encourage collaborative programs and research across institutions and locations. Governments should also provide platforms that aid the entry of start-ups into full-scale commercial bio-refineries. A combination of all of these will see lignocellulosic bio-conversion’s contribution into the fuel industry grow notably [174]. Success in producing and utilizing biofuels boosts a country’s energy security, reduces pollution, promotes research and development, affects employment as jobs are created, and provides support for the agricultural sector. Existing renewable energy and climate change policies are also expected to positively influence global adoption of bioenergy [175].

## 9. Conclusions

Bacterial bioconversion of lignocellulosic biomass has great potential, but still requires research to reveal the untapped opportunities and how they can be harnessed. Whilst genetic engineering and the advent of omics technologies offers great promise to discovering new bacterial communities yet to be studied for their lignocellulolytic ability, these processes are still largely time consuming and not cheap. Molecular studies have not eliminated the need for culture-dependent techniques, which is when an organism’s optimum functionality can be studied to enable adoption in industrial processes. More research is required to circumvent these current challenges and discover versatile, novel bacteria species which can be studied and modified for a greener outcome. Bacteria with multi-enzyme functions and extremophilic abilities will play huge roles, as they will be more versatile and able to play several roles in the production cycle. Collaborations and research-enabled environments will be vital to see a rise in bacteria adoption in lignocellulose-driven bio-refinery to derive the inherent benefits.

## Figures and Tables

**Table 1:** Recent bacterial studies using lignocellulose biomass in bio-refinery of various products.

S. N.	Bacteria Strain	Substrate (Biomass)	Degradation % (Yield)	Degradation Time	Method of Analysis	Product	References
1.	*Pantoea ananatis* *Sd-1*	Pesticide carbaryl, rice straw	45	24 h	Enzyme assays	Reducing sugars	[71]
2.	Firmicutes, Proteobacteria	Wheat, rice, sugarcane, and pea ball-milled straws	-	84 h	Biolog (MT2) microplate system	Cellulase and xylanase	[23]
3.	*Aeromonas hydrophila*, *Pseudomonas poae*, *Streptomyces thermoviolaceus*, *Klebsiella oxytoca*, *Bacillus amyloliquefaciens*	Wheat, rice, sugarcane, and pea straw	100	72 h	Enzyme assays & genomics	Strawase, cellulase	[72]
4.	*Streptomyces*, *Bacillus and Paenibacillus*	Saw dust	40–100	7–10 days	Enzyme assays & genomics	Cellulase	[73]
5.	*Opitutus terrae*, *Spirosoma linguale*, *Solibacter usitatus*	Lignite, molasses	-	5 days	Fermentation	Biogas and organic acid	[14]
6.	phosphate solubilizing (PSB) and potassium solubilizing (KSB) bacteria	Bagasse	-	7 days 28 days	Randomized blocks design and assays	Carbon Dioxide	[74]
7.	*Ochrobactrum* sp.	Palm oil mill effluent	71	6 days	Aerobic treatment	CMCase and xylanase	[75]
8.	*Pantoea ananatis* Sd-1	Rice straw	46	3 days	Enzyme assays, fenton chemistry	Biofuels	[76]
9.	*Bacillus*, *Streptomyces*, *Burkholderia*	Beech wood	-	7 days	Enzyme assays.	Xylanase	[77]
10.	*Dickeya* sp. *WS52*	Sweet Pepper and Tomato Stalk	-	4 days	Enzymatic hydrolysis and genomics	CMCase and pectinase	[78]
11.	*Clostridium* sp., *Petrimonas* sp., *Methanosarcina* sp. *and* *Methanospirillum* sp.	Palm oil mill effluent (POME)	-	18 h	PCR-DGGE and fermentation	Methane	[79]
12.	*Streptomyces lividans*	Sunflower stalks and rape straw	-	6 days	Fatty acid profiling	Triacylglycerol	[80]
13.	*Bacillus*, *Enterococcus*, *Lactococcus*, *Afipia*, *Alkaliphilus*, *Burkholderia*, *Erwinia*, *Geobacillus*, *Ralstonia*, *Rhodanobacter*, *Sediminibacterium andStreptococcus*.	Rice straw	-	4 days	Enzyme activity assay	Cellulase	[13]
14.	Firmicutes, Actinobacteria, Proteobacteria and Bacteroidetes	Molasses	-	124 days	Fermentation	Organic acids and other compounds	[81]
15.	Proteobacteria, Firmicutes and Bacteroidetes	Corn stover	-	10 days	Assays	Enzymes	[82]
16.	*Pseudomonas* sp. *GO2*.	Corn stover	99.8	131 h	Fermentation	Bioflocculant	[83]
17.	*Geobacillus* sp. *strain* *WSUCF1*	Prairie cordgrass	100	120 h	Single pot bioconversion	Biohydrogen	[84]
18.	*Streptomyces* sp. *MDS*	Rice waste biomass, wood straw, local grass powder, sugar cane barboja and sugar cane bagasse	6	6 days	Solid state fermentation	Endoglucanase, exoglucanase, cellobiases, filter pa- perase, amylase, and xylanase	[85]
19.	*Bacillus* sp. *BS-5*	Corn cob	-	72 h	Enzymes assays	Xylanase, endoglucanase	[55]
20.	*Paenibacillus, Streptomyces*	Switch grass	-	10 days	Solid-state and submerged-state cultivation	Biofuel	[86]
21.	Actinobacteria	Olive pomace	-	6 days	Submerged fermentation	Laccase, xylanase	[87]
22.	*Bacillus* sp. *K1*	Wheat Bran	44	24 h	Lipid extraction and enzyme assays	Lipid, biodiesel	[88]
23.	*Bacillus* sp. *G0*	Miscanthus	88	100 h	Pre-treatment	Xylanase	[89]
24.	*Cellulomonas*, *Pseudomonas*, *Bacillus*, *Clostridium*, *andFibrobacter*	Peat	86	42 days	Viability and decomposition tests	NA	[90]
25.	*Streptomyces* sp., *Pseudonocardiaceae*, *Micromonosporaceae*, *Saccharothrix*	Stipa and Hilaria grass biomass	-	35 days	Genomics and enzyme assays	Endo- and exo-cellulase, 27 glucosidase, endo- and exo-xylosidase and arabinofuranosidases.	[91]
26.	*Paenibacillus* sp.	Rice straw, corn straw, soybean straw, and sugarcane bagasse	-	3 days	Proximate analysis and bacteria growth comparisons	-	[92]
27.	*Bacillus cereus RSDa2, Stenotrophomonas maltophilia RSI6, Klebsiella pneumoniae RSI9*	Rice	-	45 days	Enzyme assays and metagenomics	Compost	[93]
28.	Anaerobic microbial consortium TC-5	Untreated wheat straw	45.7	9 days	Fermentation	Methane	[47]
29.	*Enterobacter xiangfangensis*, *Serratia rubidaea*, *Klebsiella pneumoniaeandCitrobacter* sp. *UWIBGS10.*	Sugarcane bagasse	-	168 h	Hydrolytic production	Glucose	[12]
30.	*Clostridium butyricum I5-42*	Starch free fibre from cassava pulp and xylan	-	24 h	Enzyme assays, fermentation	1,3-propanediol	[94]
31.	*Pantoea ananatis Sd-1*	Lignin & sugar substrates	-	24 h	Enzyme assays and genomics	Bacterial pyranose 2-oxidase (P2Ox)	[95]
32.	*Paenibacillus polymyxa ND25*	CMC, avicel, corn starch, rice straw and sugarcane bagasse	-	48 h	Enzyme assays	Endoglucanase, exoglucanase and β-glucosidase	[96]
33.	Proteobacteria, Firmicutes, Bacteroidetes Dysgonomona, Sedimentibacter, Comamonas	Energy grass	-	5–10 days	Shotgun sequencing and enzyme assays	Biogas	[44]
34.	*Pseudomonas*, *ArthrobacterandPseudoxanthomonas*	Maize stover	47	30 days	Enzyme assays and genomics	Carboxymethyl cellulase, avicelase, β-glucosidase, endo-β-1,4-xylanase, acetyl esterase, ferulic acid esterase, manganese peroxidase and laccase	[97]
35.	*Enhydrobacter*	Rice husk	-	65 days	Blackgram growth and nutrient status analysis	Compost	[98]
36.	*Bacillus velezensis 157*	Agro-industrial waste (soybean meal, wheat bran, sugarcane bagasse, wheat straw, rice husk, maize flour and maize straw)	-	72 h	Solid-state fermentation	Cellulase, xylanase, α-amylase, and pectinase	[99]
37.	*Bacteroidetes*, *Firmicutes*, *Rikenellaceae*, *Clostridiaceae*, *Porphyromonadaceae*, *Bacteroidaceae*, *Ruminococcaceae*, *Firmicutes flavefaciens*, *Ruminococcus albus*	Cow manure	-	40 days	Biochemical methane potential tests	Biogas/methane	[100]
38.	*Ethanoligenens*, *Tepidimicrobium*, *Clostridium*, *Coprococcus*, *andRuminococcus*	Swine Manure, Napier grass	36.6	21 days	Enzyme activity assay	Biogas/Methane	[101]
39.	*Prevotella*, *Bacillus*, *Thermus*, *Truepera*, and *Caldicoprobacter*, *Thermopolyspora*, *andPseudoxanthomonas*	Weed	-	-	Illumina sequencing	Compost	[102]
40.	Fibrobacter succinogenes S85	Cellulose	-	16 h	HPLC, SEM, Activity assays	Cellulases	[103]
41.	Bacteroidetes, Firmicutes, Proteobacteria, Actinobacteria, Fibrobacter, Bacteroides, Clostridium, Prevotella and Ruminococcus	Roughage	-	-	Metagenomics	Cazymes	[104]
42.	*Clostridium*, *Bacteroides*, *Ruminococcus andMethanosarcina*	Rice straw	83	30 days 126 days	Enzyme assays	Methane	[105]
43.	*Bacillus* sp. *TPF-1*	Algae, paper mill wastewater	48	72 h	Enzyme assays	CMCase and xylanase	[106]
44.	*Caldicellulosiruptor bescii*	Switch grass	80	80 h	Fermentation	Acetate	[107]
45.	*Achromobacter* sp. *(PS1)*	Rice-straw, wheat-straw and sugarcane-bagasse	73.10 and 91.13	192 h	One-factor-at-a-time approach	Biosurfactant	[108]
46.	*Lactococcus*, *Serratia*, *DysgonomonasandEnterococcus*	Bamboo shoot particles	-	6 days	Enzyme assays	Endoglucanase, β-glucosidase, xylanase, exoglucanase, laccase and lignin peroxidase	[109]
47.	Bacteroidetes, Lentisphaerae, Firmicutes and Fibrobacteres	Corn Stover	-	6 days	Gas chromatography, sequencing	Lignocellulolytic enzyme	[110]
48.	*Sphingobacterium* sp. *ksn-11*	Corn husk, peanut husk, rice bran, sugarcane bagasse, paddy straw, and coffee cherry husk	60	24 h	Sub merged fermentation and optimisation	Cellulase, xylanase, pectinase, mannanase, and laccase	[111]
49.	*Bacillus subtillis*	Palm oil mill waste	90	14 days	Batch digestion	Methane	[68]
50.	*Lactobacillus plantarum RI 11*	Molasses, rice straw, Palm Kernel Cake and soybean	-	7 days	Enzyme assays	Endoglucanase, exoglucanase, β-glucosidase and mannanase	[112]
51.	*Clostridia* sp.	Swine manure and corn stover	-	75 days	Lignocellulolytic activity assays	Methane	[113]
52.	*Clostridiales*	Wheat straw	90	93 days	Kinetics and batch assays	Methane	[114]
53.	*Parabacteroides*, *Alcaligenes*, *Lysinibacillus*, *Sphingobacterium*, *andClostridium*	Rice Straw	71	20 days	Anaerobic digestion	Endo-glucanase	[69]
54.	*Alcaligenes faecalis* subsp. *phenolicus ZY-16*	Citrus peel wastes	90	5 days	Enzyme activity assays	Bioflocculants	[70]
55.	*Clostridium thermocellum*, *C. stercorarium*, *andThermoanaerobacter thermohydrosulfuricus*	Wheat and Cattail Biomass	80	60 days	Consolidated bioprocessing	Ethanol	[115]
